# Secondary analysis of the Game of Stones trial for men with obesity: examining moderator effects and exploratory outcomes

**DOI:** 10.1002/oby.24316

**Published:** 2025-07-09

**Authors:** Stephan U. Dombrowski, Pat Hoddinott, Lisa Macaulay, Catriona O'Dolan, James Swingler, Seonaidh Cotton, Alison Avenell, Abraham M. Getaneh, Cindy Gray, Kate Hunt, Frank Kee, Alice MacLean, Michelle C. McKinley, Claire Torrens, Katrina Turner, Marjon van der Pol, Graeme MacLennan

**Affiliations:** ^1^ Faculty of Kinesiology University of New Brunswick Fredericton New Brunswick Canada; ^2^ Nursing, Midwifery and Allied Health Professions Research Unit University of Stirling Stirling UK; ^3^ Health Services Research Unit University of Aberdeen Aberdeen UK; ^4^ Centre for Healthcare Randomised Trials University of Aberdeen Aberdeen UK; ^5^ Health Economics Research Unit University of Aberdeen Aberdeen UK; ^6^ School of Social and Political Sciences University of Glasgow Glasgow UK; ^7^ Institute for Social Marketing and Health University of Stirling Stirling UK; ^8^ Centre for Public Health Queen's University Belfast Belfast UK; ^9^ Centre for Academic Primary Care University of Bristol Bristol UK

## Abstract

**Objective:**

The objective was to explore whether socioeconomic, health, and behavioral characteristics moderate the effectiveness of a text message intervention with or without financial incentives versus a control group and to examine differences in exploratory outcomes.

**Methods:**

This three‐group randomized trial including 585 men with obesity compared daily automated behavioral text messages alongside financial incentives, text messages alone, and a waiting list control for 12 months. Moderator analyses examined percentage weight change after 12 months for 9 socioeconomic and 11 health factors. Exploratory outcomes included the following: self‐reported physical activity, sedentary behavior, smoking and alcohol behaviors, engagement in 15 weight‐management strategies, and weight‐management–related confidence.

**Results:**

No moderator effects were found by any factors for either comparison versus control. There were no differences across groups for health behaviors. The texts with incentives group had higher levels of engagement in six strategies including weight goals, food changes, and self‐weighing and higher levels of confidence compared with the control.

**Conclusions:**

The Game of Stones interventions were equally effective across various subgroups based on socioeconomic, health, or well‐being status. Texts with financial incentives group participants showed better engagement for some intervention elements. The implementation of Game of Stones is unlikely to increase health inequalities. Future studies should focus on increasing engagement.

## INTRODUCTION

Obesity is increasingly a worldwide problem and elevates the risk of adverse health conditions for individuals carrying excess fat on their body [1]. Although approximately 26% of UK and 43% of US adult men are estimated to be living with obesity [2, 3], evidence suggests that men are less likely than women to engage in weight‐management interventions, programs, and services [4]. Gender‐sensitized behavioral interventions that take the diverse needs, experiences, and perspectives of men into account are needed [5].

Behavioral weight‐management interventions seeking volitional changes in eating behaviors and physical activity remain a cornerstone of accessible, low‐risk obesity treatment. Several behavioral weight‐management interventions for men have started to successfully engage men and support their weight loss [6, 7]. For example, interventions delivered at professional sport team locations have successfully supported weight loss in men through behavioral weight‐management interventions. These included diverse sporting contexts, such as football [8], rugby [9], and hockey [10]. However, not all men are able to attend group‐delivered and in‐person interventions. As part of a whole system approach to obesity management, additional interventions that are inclusive and a minimal burden for participants and clinical staff are needed.

Text message–delivered behavioral interventions are a scalable, inclusive, and low‐burden form of delivery [11] and show effectiveness to support weight loss. A systematic review that included 12 randomized controlled trials reported that text messaging–based weight‐loss interventions showed a mean difference in weight change of −2.3 (95% confidence interval [CI]: −3.2 to −1.4) kg compared with control [12]. However, evidence is limited in terms of representation of men, and few interventions report results at 12 months or longer. Financial incentives can help men living with obesity lose weight [13], and the addition of behavior‐change techniques and consideration of economic theory can enhance effectiveness [14, 15]. Financial incentives have been shown to support clinically significant weight loss in low‐income populations living with obesity [16].

The Game of Stones trial randomized 585 men with obesity to behavioral text messages with financial incentives, text messages alone, or a waiting list control group [17]. Findings showed a 4.8% weight loss at 12 months in participants who received text messages with financial incentives, which was significantly different from the control group who lost 1.3% of their baseline weight [18]. The texts alone group lost 2.7%, which was not significantly different to the control group. Game of Stones is a remotely delivered, low‐burden intervention with direct, in‐person contact limited to four brief weight assessments over 12 months, making it potentially relevant for service providers serving men interested in self‐directed weight loss supported through text messages with incentives.

Although the Game of Stones trial results are positive overall, the intervention outcomes require further examination. It is critical to ensure that the intervention does not disproportionately affect vulnerable subgroups, such as those disadvantaged by socioeconomic circumstances or health. Evidence gaps remain for engagement and effectiveness of weight‐management interventions in men living with obesity who also report low socioeconomic status [19, 20]. A systematic review examining socioeconomic factors in 36 trials of behavioral weight‐loss interventions for men found that only one study examined potential differential intervention effects across socioeconomic groups [21]. Moreover, to effectively target individuals who are most likely to benefit from an intervention, it is critical to examine differential effectiveness in relevant subgroups, including characteristics such as relationship or employment status.

In addition to examining overall intervention effectiveness on primary and secondary outcomes, exploratory outcome may provide additional insights into the impact on intervention processes. For behavioral weight‐management interventions, process‐related outcomes, such as behavior changes, engagement in weight‐management strategies, and psychological cognitions, can clarify the effect an intervention had on weight‐change–related factors.

Equitable, inclusive, low‐burden, and scalable interventions are required to address health inequalities associated with obesity and engage underserved populations. It is important for behavioral interventions to avoid intervention‐generated inequalities and contribute toward improving obesity‐related health at a population level [22]. Moreover, to fully understand the impact of behavioral weight‐management interventions, changes in process outcomes, such as behavior, engagement in weight‐management strategies, and psychological cognitions, are required. Therefore, this secondary analysis of the Game of Stones trial aims to explore whether baseline socioeconomic, health, and well‐being characteristics moderate the effectiveness of the primary outcome of percentage weight change at 12 months for men with obesity randomized to a text message intervention with or without financial incentives versus a control group, and to examine differences in exploratory outcomes.

## METHODS

### Intervention

The Game of Stones trial was a three‐arm, parallel group, assessor‐blinded randomized clinical trial conducted between July 2021 and July 2023 in three UK areas: Belfast, Bristol, and Glasgow [17, 18]. Men were invited through family practices, community information, and social media targeting disadvantaged areas. Overall, 585 men with body mass index (BMI) ≥30 kg/m^2^ were recruited.

The three study arms were as follows: 1) daily automated behavior‐focused text messages designed to support weight management for 12 months alongside loss‐framed incentives in which money was “lost” from an initial endowment of $490 (£400) by not meeting verified weight‐loss targets (5% at 3 months, 10% at 6 months, and maintaining 10% weight loss at 12 months) in comparison with baseline weight; 2) text messages (as described in 1) alone; or 3) a 12‐month waiting list for 3 months of text messages. All groups received access to a website containing evidence‐based weight‐management information and a pedometer at baseline. Intervention groups also received localized web pages signposting to services and self‐monitoring web pages.

The study received ethical approval from the North of Scotland Research Ethics Committee 2 (20/NS/0141), and the protocol has been published [17]. A participant flow diagram also has been published [18].

### Outcomes and assessments

Outcomes and assessments were based on the Game of Stones feasibility trial [23], which included extensive public, patient, and stakeholder involvement to assess acceptability and burden of data collection tools informed by guidance on outcomes in weight‐management trials (standardised reporting of lifestyle weight management interventions to aid evaluation [STAR‐LITE] [24]), PROGRESS (i.e., place of residence, race/ethnicity/culture/language, occupation, gender/sex, religion, education, socioeconomic status, and social capital)‐Plus characteristics [25], and Consolidated Standards of Reporting Trials (CONSORT) equity reporting guidance [26]. The study balanced potential academic and participant benefits and harms of data collection [27].

Baseline data were collected before randomization, and previously piloted [23] and validated measures were used, as available. No consensus on the most appropriate measures to evaluate behavioral weight‐management interventions in men with obesity currently exists. Outcomes were selected considering the different study recruitment routes of community and primary care. Participants included both men who were not engaging in health services as well as those with multiple long‐term conditions and/or a disability.

Prespecified subgroup analyses for moderators of the primary outcome of percentage weight change at 12 months from baseline were undertaken within three categories: 1) socioeconomic factors; 2) health and well‐being status; and 3) recruitment route.

#### Socioeconomic factors

The assessments of level of disadvantage included use of the Index of Multiple Deprivation (IMD), which is a measure of relative deprivation based on the UK postcode address where participants live, drawing on variables such as income, education, and crime rates. The IMD can be used to divide the population into five deprivation categories, which, for the current analysis, were aggregated into the two more deprived categories compared with the three more affluent categories. Data from England, Scotland, and Northern Ireland were classified as per the country‐specific methodology for allocation of IMD subgroup classification [25, 28].

Guidance published by the UK Office for National Statistics [29] was used to harmonize and score key individual level variables, including (participant) education (university degree level or above vs. other qualification vs. no qualification), living status (living alone vs. living with others), and relationship status (single vs. married/in a partnership). The harmonized guidance from the Scottish Government [30] was used to assess working status (in paid work/self‐employed vs. unpaid).

Perceived wealth was assessed using three items [31] (“I feel that I have enough money,” “I feel that I live in a relatively wealthy neighbourhood,” and “I feel relatively wealthy compared to others”), which were scored from 0 (strongly agree) to 100 (strongly disagree) and dichotomized into low (≤50) and high (≥51). The perceived wealth measures were unintentionally reverse scored, with lower scores indicating higher perceived wealth, unlike the original measure in which higher scores indicate higher perceived wealth.

Financial strain was assessed using one item based on French (2017) [32] (“How well would you say you yourself are managing financially these days?”), with five possible response options, dichotomized into easier (“living comfortably,” “doing alright,” and “just about getting by”) versus harder (“finding it quite difficult” and “finding it very difficult”).

#### Health and well‐being factors

Quality of life was assessed using the five‐level EuroQol‐5 Dimension scale (EQ‐5D‐5L) overall utility score (dichotomized into high [>0.4005] vs. low [≤0.4005]) and EQ‐5D‐5L Anxiety and Depression dimension (dichotomized into low [1, 2, 3] vs. high [4, 5]) [33]. The EQ‐5D‐5L has excellent psychometric properties across a broad range of populations, conditions, and settings [34].

Mental well‐being was assessed using the Warwick‐Edinburgh Mental Well‐Being Scale (WEMWBS) [35] consisting of 14 items (e.g., “I've been feeling optimistic about the future”) scored from 1 (none of the time) to 5 (all of the time), dichotomized into low (≤40) versus high (≥41). WEMWBS has been shown to be a psychometrically robust scale [35].

Mental health was assessed using four items of the reliable and validated Patient Health Questionnaire‐4 (PHQ‐4) [36], consisting of an anxiety subscale (Generalized Anxiety Disorder‐2 [GAD‐2], two items) and a depression subscale (Patient Health Questionnaire‐2 [PHQ‐2], two items). Items were scored from 0 (not at all) to 3 (nearly every day) and summed and dichotomized into high (≥3 for GAD‐2 or PHQ‐2) versus low (≤2 for GAD‐2 or PHQ‐2).

Perceived weight‐related stigma was assessed using the reliable and validated Weight Self‐Stigma Questionnaire [37] consisting of 12 items (e.g., “I feel guilty because of my weight problems”) scored from 0 (completely disagree) to 5 (completely agree) and dichotomized into high (≥42) versus low (≤41).

Comorbidities were assessed with the item “Has a doctor ever told you that you have/had…?” followed by the response options “a stroke (including ministroke),” “high blood pressure,” “a heart condition such as angina or atrial fibrillation,” “diabetes,” “cancer,” “arthritis,” and “a mental health condition” (dichotomized into yes for those reporting at least one comorbidity vs. no for those reporting none). The presence of multiple long‐term conditions was defined as the coexistence of two or more comorbidities. In addition, a self‐reported mental health condition (yes vs. no) and diabetes (yes vs. no) were analyzed separately in subgroup analyses.

A variable labeled “Possible Latent Mental Health Condition” was defined for men who did not self‐report a mental health condition but whose scores on at least one of the PHQ‐4, EQ‐5D‐5L Anxiety and Depression dimension, WEMWBS, or Weight Self‐Stigma Questionnaire exceeded a threshold suggesting a possible undetected mental health condition (see previous paragraphs for scoring details).

Self‐reported disability was assessed with the two items based on Office for National Statistics definitions [38]: “Do you have any physical or mental health conditions or illnesses lasting or expected to last 12 months or more?” and “Do any of your conditions or illnesses reduce your ability to carry out day‐to‐day activities?” Those answering yes to both were defined as having a disability.

Alcohol consumption was measured using a single question (“During the last month, how many days did you usually have any kind of drink containing alcohol?”), with eight possible response options ranging from “never” to “every day” (dichotomized into drinking every day vs. not every day).

#### Recruitment

Participants were categorized according to the route of recruitment (community based vs. via general practice).

#### Secondary exploratory outcomes

Physical activity and sedentary behavior were assessed from the self‐reported number of days of vigorous and moderate physical activity and time spent sitting, respectively, using the International Physical Activity Questionnaire [39].

Smoking status was measured with one item (“Do you currently smoke or have you ever smoked?”), with response options “yes, I currently smoke every day,” “yes, I currently smoke, but not every day,” “yes, I used to smoke but have quit,” and “no, I have never smoked.”

Self‐monitoring of activity and weight were assessed with one item, respectively (“How often do you monitor your steps?” and “How often do you keep track of your weight by weighing yourself?”), with six response options ranging from “never” to “every day.”

Weight‐management strategies were assessed with the following item: “Which of these strategies have you used in the last 12 months to lose weight?” Participants were provided with 13 response options (e.g., “had a weight goal to work towards”) based on evidence of effective strategies for weight management [40].

Confidence in ability to lose weight and confidence in ability to maintain weight loss long term were each assessed with a single item (“How confident are you in your ability to lose weight?” and “How confident are you in your ability to keep lost weight off in the long term?”), with responses on a 7point scale ranging from 1 (not confident) to 7 (very confident).

#### Sample size calculation

The sample size calculation for this trial was for the primary outcome of percentage weight change from baseline to 12 months [18].

#### Analysis

The primary outcome subgroup modeling used linear regression adjusted for the recruitment areas (Belfast, Bristol, Glasgow) and recruitment route (family practice or community), treatment group, the subgroup of interest, and a treatment‐by‐subgroup interaction term. CI values are presented at 99.5% to reflect the number of subgroups tested and the exploratory nature of analysis, equivalent to a stringent level of evidence required for significance of *p* < 0.005. Results are summarized as forest plots of within‐subgroup treatment effects and the interaction term testing the moderating effect of the subgroup.

Subgroup analyses are split into confirmatory and exploratory. Confirmatory subgroup analyses (as prespecified in the statistical analysis plan) included obesity‐related comorbidity (present vs. absent) and diabetes (present vs. absent). The confirmatory subgroup analyses are based on hypothesized directions of effect modification of the interventions informed by the weight‐loss literature [41]. Weight loss and/or weight‐loss maintenance are part of disease management for many obesity‐related comorbidities, for example, diabetes and cardiovascular disease. All other prespecified subgroup analyses were designated as exploratory.

Secondary exploratory outcomes were analyzed using a generalized linear model suitable for the outcome distribution, adjusting for recruitment center, recruitment route, and the baseline measure of the outcome if measured. CI values for all secondary outcomes are presented at 97.5% for all secondary outcomes.

All analyses were conducted as a complete case analysis and did not impute for missingness in line with the prespecified statistical analysis plan.

High levels of missingness in the subgroup analyses were accounted for by introducing a missing group if the missing element of a variable exceeded 10% of the total responses.

## RESULTS

A total of 585 participants were randomized to the texts with financial incentives group (*n* = 196), the texts alone group (*n* = 194), or the waiting list control group (*n* = 195), and 73% of participants (*n* = 426) provided weight data at 12 months.

Key baseline characteristics are reported in Table 1. Intervention groups were comparable across trial groups (for information on all assessed baseline characteristics see Hoddinott et al. [18]). Participants had a mean BMI of 37.7 (SD 5.7) and a mean age of 50.7 (SD 13.3) years, with participants' ages ranging from 19 to 84 years. Most were of White ethnicity (93%), were married/living with a partner (62%), and reported one or more comorbidities (71%), including 18% of participants overall reporting diabetes.

**TABLE 1:** Baseline characteristics by treatment allocation.

	Texts with incentives (*n* = 196)	Texts alone (*n* = 194)	Waiting list (*n* = 195)
Age, mean (SD), y; *n*	50.0 (12.7); 195	51.7 (13.3); 194	50.2 (13.9); 195
Weight and BMI, mean (SD)	*n* = 196	*n* = 194	*n* = 195
Weight, kg	120.3 (20.1)	117.2 (17.9)	118.1 (21.6)
BMI, kg/m^2^	38.2 (5.9)	37.3 (4.7)	37.8 (6.4)
Deprivation category, *n* (%)	*n* = 195	*n* = 192	*n* = 194
Most deprived	48 (25)	36 (19)	50 (26)
More deprived	28 (14)	37 (19)	28 (14)
Deprived	25 (13)	33 (17)	29 (15)
Less deprived	39 (20)	40 (21)	31 (16)
Least deprived	55 (28)	46 (24)	56 (29)
Ethnic Group, *n* (%)	*n* = 190	*n* = 186	*n* = 188
Asian/Asian British	2 (1.1)	3 (1.6)	6 (3.2)
Black/African/Caribbean/Black British	3 (1.6)	3 (1.6)	3 (1.6)
Mixed/multiple ethnic groups	2 (1.1)	‐	4 (2.1)
White	179 (94)	174 (94)	172 (92)
Other	3 (1.6)	3 (1.6)	2 (1.1)
Prefer not to say	1 (0.5)	3 (1.6)	1 (0.5)
Comorbidities, *n* (%)	*n* = 196	*n* = 193	*n* = 194
One or more comorbidities	136 (69)	136 (70)	144 (74)
MLTC	82 (42)	82 (42)	71 (36)
Physical or mental disability, *n* (%)	*n* = 193	*n* = 193	*n* = 192
Disability	60 (31)	47 (24)	58 (30)
Highest educational qualification, *n* (%)	*n* = 182	*n* = 166	*n* = 174
Degree level or above	92 (51)	71 (43)	86 (49)
Another kind of qualification	90 (49)	95 (57)	88 (51)

Abbreviation: MLTC, multiple long‐term conditions.

The main results have been published previously [18]. The overall mean (SD) percentage weight change was −4.8% (6.1%) for the texts with financial incentives group, −2.7% (6.3%) for the texts alone group, and −1.3% (5.5%) for the control group. At the 12‐month follow‐up, the texts with incentives group had significantly greater weight loss (mean difference in percentage change from baseline, −3.2%; 97.5% CI: −4.6% to −1.9%; *p* < 0.001), and the texts alone group did not have significantly greater weight loss (mean difference in percentage change from baseline, −0.4%; 97.5% CI: −2.9% to 0.0%; *p* = 0.05) compared with the control group.

### Moderator analyses

**FIGURE 1:**
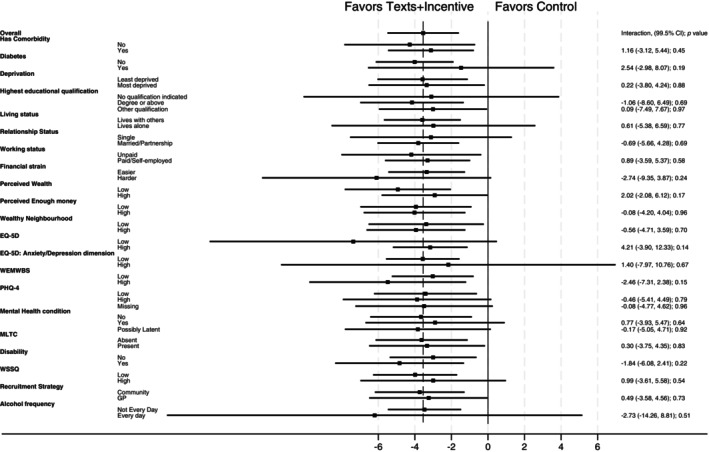
Forest plot for subgroup analysis comparing percentage weight loss (99.5% CI) at 12 months from baseline between the texts with incentives group and the control group. EQ‐5D, EuroQol‐5 Dimension scale; MLTC, multiple long‐term conditions; PHQ‐4, Patient Health Questionnaire‐4; WEMWBS, Warwick‐Edinburgh Mental Well‐Being Scale; WSSQ, Weight Self‐Stigma Questionnaire.

**FIGURE 2:**
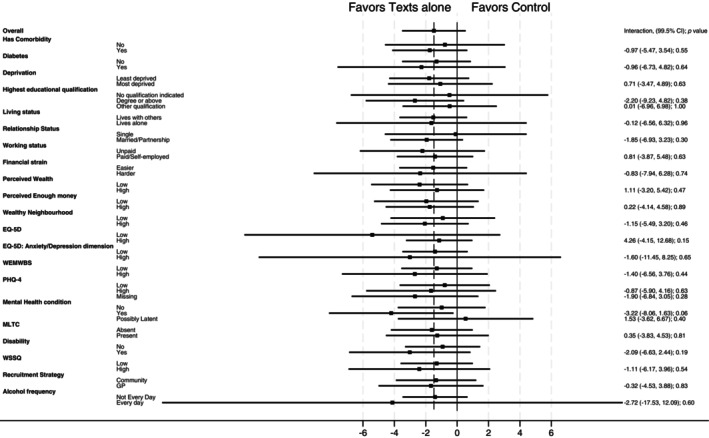
Forest plot for subgroup analysis comparing percentage weight loss (99.5% CI) at 12 months from baseline between the texts alone group and the control group. EQ‐5D, EuroQol‐5 Dimension scale; MLTC, multiple long‐term conditions; PHQ‐4, Patient Health Questionnaire‐4; WEMWBS, Warwick‐Edinburgh Mental Well‐Being Scale; WSSQ, Weight Self‐Stigma Questionnaire.

Confirmatory subgroup analyses found no evidence for an interaction for the presence of a comorbidity or diabetes for either the texts with incentives compared with the control group or the texts alone group compared with the control group (*p* values for interactions ≥ 0.19; Table 2; Figures 1, 2).

**TABLE 2:** Subgroup analyses for percentage weight change at 12 months from baseline.

Analysis type	Texts with incentives (*n* = 146)	Texts alone (*n* = 128)	Control (*n* = 152)	Texts with incentives vs. control	Texts alone vs. control
Subgroup category^a^				Interaction effect, mean difference (99.5% CI); *p* value	Interaction effect, mean difference (99.5% CI); *p* value
Confirmatory analyses: weight change, mean (SD), %; *n*					
Has comorbidity					
No	−5.8 (5.3); 46	−2.4 (6.6); 36	−1.7 (6.4); 43		
Yes	−4.3 (6.5); 99	−2.9 (6.2); 92	−1.1 (5.1); 109	1.16 (−3.12 to 5.44); 0.45	−0.97 (−5.47 to 3.54); 0.55
Has diabetes					
No	−5.3 (6.0); 117	−2.6 (6.5); 106	−1.3 (5.7); 134		
Yes	−2.8 (6.7); 28	−3.5 (5.3); 22	−1.3 (3.8); 18	2.54 (−2.98 to 8.07); 0.19	−0.96 (−6.73 to 4.82); 0.64
Exploratory analyses: weight change, mean (SD), %; *n*					
Socioeconomic factors					
Deprivation category					
Less deprived	−4.3 (5.9); 90	−2.4 (6.9); 80	−0.7 (5.0); 95		
More deprived	−5.6 (6.6); 56	−3.4 (5.0); 47	−2.3 (6.1); 56	0.22 (−3.80 to 4.24); 0.88	0.71 (−3.47 to 4.89); 0.63
Highest educational qualification					
No qualification indicated	−5.6 (7.3); 10	−3.0 (5.3); 15	−2.6 (4.1); 14		
Degree or above	−4.6 (5.8); 70	−3.0 (6.8); 49	−0.4 (5.3); 73	−1.06 (−8.60 to 6.49); 0.69	−2.20 (−9.23 to 4.82); 0.38
Other qualification	−4.9 (6.4); 66	−2.5 (6.1); 64	−2.0 (5.8); 65	0.09 (−7.49 to 7.67); 0.97	0.01 (−6.96 to 6.98); 1.00
Living status					
Lives with others	−4.6 (6.2); 127	−2.5 (6.4); 115	−1.0 (5.4); 133		
Lives alone	−5.8 (5.8); 18	−4.5 (4.8); 13	−2.9 (5.6); 19	0.61 (−5.38 to 6.59); 0.77	−0.12 (−6.56 to 6.32); 0.96
Relationship status					
Single	−5.0 (7.0); 28	−1.8 (4.0); 26	−1.9 (6.0); 31		
Married/partnership	−4.8 (5.9); 116	−3.0 (6.8); 101	−1.0 (5.3); 116	−0.69 (−5.66 to 4.28); 0.69	−1.85 (−6.93 to 3.23); 0.30
Working status					
Not in paid employment	−6.0 (6.5); 40	−4.0 (5.5); 34	−1.7 (4.9); 38		
Paid/self‐employed	−4.3 (5.9); 103	−2.4 (6.6); 89	−1.1 (5.7); 109	0.89 (−3.59 to 5.37); 0.58	0.81 (−3.87 to 5.48); 0.63
Financial strain					
Easier	−4.4 (6.0); 127	−2.6 (6.3); 114	−1.1 (5.3); 131		
Harder	−8.3 (6.6); 15	−4.5 (6.8); 11	−2.1 (7.2); 14	−2.74 (−9.35 to 3.87); 0.24	−0.83 (−7.94 to 6.28); 0.74
Perceived wealth					
Low	−5.4 (6.2); 77	−2.8 (5.4); 61	−0.5 (4.7); 64		
High	−4.3 (6.1); 64	−2.7 (7.5); 57	−1.4 (6.1); 76	2.02 (−2.08 to 6.12); 0.17	1.11 (−3.20 to 5.42); 0.47
Perceived enough money					
Low	−4.9 (6.7); 71	−2.9 (6.0); 50	−1.0 (4.6); 58		
High	−4.9 (5.7); 70	−2.7 (6.8); 67	−0.9 (5.9); 83	−0.08 (−4.20 to 4.04); 0.96	0.22 (−4.14 to 4.58); 0.89
Perceived wealth compared with neighborhood					
Low	−4.9 (6.4); 66	−2.5 (4.9); 52	−1.6 (4.5); 55		
High	−4.8 (6.0); 75	−2.9 (7.4); 68	−0.9 (6.2); 88	−0.56 (−4.71 to 3.59); 0.70	−1.15 (−5.49 to 3.20); 0.46
Health and well‐being factors					
EQ‐5D‐5L					
Low	−7.3 (8.6); 14	−5.3 (7.4); 11	0.2 (6.4); 7		
High	−4.5 (5.8); 131	−2.5 (6.1); 117	−1.4 (5.5); 142	4.21 (−3.90 to 12.33); 0.14	4.26 (−4.15 to 12.68); 0.15
EQ‐5D‐5L: Anxiety/Depression dimension					
Low	−4.9 (6.1); 139	−2.7 (6.2); 123	−1.3 (5.4); 144		
High	−3.1 (8.4); 6	−3.6 (9.1); 5	−0.7 (6.8); 8	1.40 (−7.97 to 10.76); 0.67	−1.60 (−11.45 to 8.25); 0.65
WEMWBS					
Low	−4.4 (6.1); 107	−2.6 (6.3); 103	−1.4 (5.7); 121		
High	−6.1 (6.1); 35	−3.2 (6.2); 25	−0.4 (4.2); 28	−2.46 (−7.31 to 2.38); 0.15	−1.40 (−6.56 to 3.76); 0.44
PHQ‐4					
Low	−4.8 (6.2); 67	−2.2 (6.4); 62	−1.5 (5.3); 80		
High	−4.9 (6.3); 37	−2.6 (6.1); 34	−0.9 (5.5); 33	−0.46 (−5.41 to 4.49); 0.79	−0.87 (−5.90 to 4.16); 0.63
Missing^a^	−4.7 (6.0); 42	−3.9 (6.3); 32	−1.2 (5.8); 39	−0.08 (−4.77 to 4.62); 0.96	−1.90 (−6.84 to 3.05); 0.28
Mental health condition					
No	−5.2 (5.9); 68	−2.5 (4.1); 67	−1.6 (5.2); 79		
Yes	−3.2 (6.6); 38	−4.4 (8.0); 33	−0.3 (6.0); 40	0.77 (−3.93 to 5.47); 0.64	−3.22 (−8.06 to 1.63); 0.06
Possibly latent	−5.7 (5.9); 39	−1.3 (7.8); 28	−1.7 (5.3); 33	−0.17 (−5.05 to 4.71); 0.92	1.53 (−3.62 to 6.67); 0.40
MLTC					
Absent	−4.8 (6.1); 82	−2.8 (7.2); 73	−1.2 (5.8); 100		
Present	−4.8 (6.2); 63	−2.7 (4.9); 55	−1.4 (4.7); 52	0.30 (−3.75 to 4.35); 0.83	0.35 (−3.83 to 4.53); 0.81
Disability					
No	−4.6 (6.0); 98	−2.5 (6.4); 95	−1.6 (5.4); 105		
Yes	−5.3 (6.6); 46	−3.4 (5.8); 33	−0.4 (5.5); 46	−1.84 (−6.08 to 2.41); 0.22	−2.09 (−6.63 to 2.44); 0.19
Weight stigma (WSSQ)					
Low	−5.3 (6.3); 101	−2.6 (4.2); 102	−1.3 (5.2); 116		
High	−4.0 (5.7); 42	−3.4 (11.6); 25	−0.9 (6.3); 32	0.99 (−3.61 to 5.58); 0.54	−1.11 (−6.17 to 3.96); 0.54
Alcohol frequency					
Not every day	−4.8 (6.2); 140	−2.7 (6.3); 125	−1.3 (5.5); 146		
Every day	−4.6 (4.6); 5	−2.1 (1.3); 2	1.9 (2.5); 4	−2.73 (−14.26 to 8.81); 0.51	−2.72 (−17.53 to 12.09); 0.60
Recruitment route					
Recruitment					
Community	−5.0 (6.0); 88	−2.5 (6.2); 76	−1.2 (6.0); 102		
GP	−4.6 (6.4); 58	−3.0 (6.4); 52	−1.4 (4.1); 50	0.49 (−3.58 to 4.56); 0.73	−0.32 (−4.53 to 3.88); 0.83

^a^ A missing subgroup was created if the missing element of a variable exceeded 10% of the total responses; subgroup analyses for social weight loss are displayed in Table S2.

Abbreviations: EQ‐5D‐5L, EuroQol‐5 Dimension; GP, general practice; MLTC, multiple long‐term conditions; PHQ‐4, Patient Health Questionnaire‐4; WEMWBS, Warwick‐Edinburgh Mental Well‐Being Scale; WSSQ, Weight Self‐Stigma Questionnaire.

Exploratory subgroup analyses for socioeconomic factors found no evidence for an interaction for deprivation category, education, living status, relationship status, working status, financial strain, perceived wealth, perceived enough money, and perceived neighborhood wealth for either the texts with incentives group compared with the control group or the texts alone group compared with the control group (*p* values for interactions ≥ 0.02; Table 2; for subgroup analyses examining change in financial strain, perceived wealth, perceived enough money, and perceived wealth compared with neighborhood, see Table S1).

Exploratory subgroup analyses for health and well‐being status found no evidence for an interaction for overall quality of life (EQ‐5D‐5L), anxiety/depression (EQ‐5D‐5L dimensions), mental well‐being (WEMWBS), mental health (PHQ‐4), self‐reported mental health condition, multiple long‐term conditions, disability status, weight stigma, and alcohol consumption for either the texts with incentives compared with the control group or the texts alone group compared with the control group (*p* values for interactions ≥ 0.06; Table 2; for subgroup analyses examining change in social weight loss reported by participants at 12 months, see Table S1).

Exploratory subgroup analyses for recruitment route found no evidence for an interaction for either the texts with incentives compared with the control group or the texts alone group compared with the control group (*p* value for interactions ≥ 0.73; Table 2).

### Secondary outcomes

#### Health behaviors

There were no statistically significant differences in self‐reported number of days of vigorous and moderate physical activity or time spent sedentary between either the texts with incentives or the texts alone groups and the control group (Table 3). Moreover, no statistically significant differences were found for alcohol consumption and smoking status between either intervention group and the control group.

**TABLE 3:** Health behaviors by treatment allocation at baseline and 12 months.

Variables	Baseline			12 mo			Texts with incentives vs. control (97.5% CI)	Texts alone vs. control (97.5% CI)
	Texts with incentives (*n* = 195)	Texts alone (*n* = 193)	Control (*n* = 193)	Texts with incentives (*n* = 143)	Texts alone (*n* = 127)	Control (*n* = 151)		
Vigorous physical activity in past wk^a^, mean (SD), d; *n*	1.2 (1.6); 193	1.3 (1.9); 192	1.1 (1.7); 191	1.6 (2.0); 144	1.5 (1.8); 127	1.4 (1.8); 151	0.3 (−0.2 to 0.7)	0.1 (−0.4 to 0.5)
Change in vigorous physical activity from baseline (d), *n*/*N* (%)								
Decreased				30/141 (21.3)	26/126 (20.6)	31/148 (20.9)		
Stayed the same				58/141 (41.1)	53/126 (42.1)	71/148 (48.0)		
Increased				53/141 (37.6)	47/126 (37.3)	46/148 (31.1)		
Moderate physical activity in past wk^a^, mean (SD), d; *n*	3.4 (2.2); 194	3.2 (2.3); 192	3.3 (2.3); 193	3.8 (2.3); 144	3.3 (2.3); 128	3.5 (2.2); 152	0.4 (−0.1 to 0.9)	−0.0 (−0.6 to 0.5)
Change in moderate physical activity from baseline (d), *n*/*N* (%)								
Decreased				42/142 (29.6)	45/127 (35.4)	53/150 (35.3)		
Stayed the same				31/142 (21.8)	40/127 (31.5)	38/150 (25.3)		
Increased				69/142 (48.6)	42/127 (33.1)	59/150 (39.3)		
Sedentary behavior in past wk^a^, mean (SD), d; *n*	0.6 (0.3); 191	0.7 (0.3); 191	0.7 (0.3); 192	0.6 (0.3); 142	0.7 (0.3); 128	0.6 (0.3); 151	0.0 (−0.0 to 0.1)	0.0 (−0.0 to 0.1)
Change in sedentary behavior from baseline (d), *n*/*N* (%)								
Decreased				70/138 (50.7)	69/127 (54.3)	76/149 (51.0)		
Stayed the same				9/138 (6.5)	14/127 (11.0)	16/149 (10.7)		
Increased				59/138 (42.8)	44/127 (34.6)	57/149 (38.3)		
Frequency of alcohol consumption in past mo^b^, *n*/*N* (%)							0.8 (0.5 to 1.4)	1.1 (0.6 to 1.8)
Every day	6/195 (3.1)	3/192 (1.6)	6/193 (3.1)	7/143 (4.9)	4/127 (3.1)	3/151 (2.0)		
5–6 times/wk	12/195 (6.2)	7/192 (3.6)	7/193 (3.6)	9/143 (6.3)	3/127 (2.4)	6/151 (4.0)		
3–4 times/wk	24/195 (12.3)	30/192 (15.6)	30/193 (15.5)	18/143 (12.6)	14/127 (11.0)	20/151 (13.2)		
Twice/wk	33/195 (16.9)	37/192 (19.3)	36/193 (18.7)	22/143 (15.4)	25/127 (19.7)	28/151 (18.5)		
Once/wk	20/195 (10.3)	27/192 (14.1)	29/193 (15.0)	12/143 (8.4)	19/127 (15.0)	23/151 (15.2)		
2–3 times/mo	31/195 (15.9)	21/192 (10.9)	20/193 (10.4)	21/143 (14.7)	15/127 (11.8)	22/151 (14.6)		
Once/mo	18/195 (9.2)	21/192 (10.9)	26/193 (13.5)	18/143 (12.6)	13/127 (10.2)	24/151 (15.9)		
Never	51/195 (26.2)	46/192 (24.0)	39/193 (20.2)	36/143 (25.2)	34/127 (26.8)	25/151 (16.6)		
Change in alcohol consumption from baseline, *n*/*N* (%)								
Decreased				35/142 (24.6)	40/126 (31.7)	48/149 (32.2)		
Stayed the same				82/142 (57.7)	65/126 (51.6)	80/149 (53.7)		
Increased				25/142 (17.6)	21/126 (16.7)	21/149 (14.1)		
Smoking status (current)^b^, *n*/*N* (%)							1.4 (0.6 to 3.4)	1.2 (0.5 to 3.0)
Current smoker (regular)	14/194 (7.2)	8/193 (4.1)	9/191 (4.7)	8/143 (5.6)	4/126 (3.2)	6/151 (4.0)		
Current smoker (irregular)	7/194 (3.6)	7/193 (3.6)	5/191 (2.6)	5/143 (3.5)	‐	5/151 (3.3)		
Ex‐smoker	67/194 (34.5)	88/193 (45.6)	68/191 (35.6)	48/143 (33.6)	63/126 (50.0)	55/151 (36.4)		
Never smoked	106/194 (54.6)	90/193 (46.6)	109/191 (57.1)	82/143 (57.3)	59/126 (46.8)	85/151 (56.3)		
Change in smoking status from baseline, *n*/*N* (%)								
Decreased				6/142 (4.2)	7/126 (5.6)	5/147 (3.4)		
Stayed the same				134/142 (94.4)	116/126 (92.1)	136/147 (92.5)		
Increased				2/142 (1.4)	3/126 (2.4)	6/147 (4.1)		

^a^ Scores range from 0 to 7 (none to every day). Each outcome was analyzed using an adjusted linear model.

^b^ Drinking scores range from 1 to 8 (every day to never), and smoking scores range from 1 to 4 (yes, every day to no, never). Both outcomes analyzed using an ordered logit model adjusting for baseline scores.

#### Weight‐management strategies

Nine of the fifteen measured weight‐loss strategies (Table 4) investigated showed no differences between the texts with incentives and control groups. Compared with the control group, participants in the texts with incentives group were more likely to report self‐weighing (odds ratio [OR] 2.2 [97.5% CI: 1.3–3.5]; Table 4). At 12 months, 56.8% of the texts with incentives group reported self‐monitoring their weight at least once a week, compared with 37.7% in the control group. There was no difference in self‐weighing between the texts alone and control groups. Moreover, there was no difference in self‐monitoring pedometer steps between the control group and either the texts with incentives or texts alone groups. Compared with the control group, participants in the texts with incentives group were more likely to report avoiding certain foods (OR 3.0 [97.5% CI: 1.6–5.7]), having a weight goal to work toward (OR 4.7 [97.5% CI: 2.6–8.5]), reminding oneself of the reasons for trying to lose weight (OR 3.2 [97.5% CI: 1.8–5.8]), swapping one type of food for another (OR 2.1 [97.5% CI: 1.2–3.6]), and telling others about weight‐loss goals (OR 3.9 [97.5% CI: 2.2–7.1]). At 12 months, 65.5% of the texts with incentives group participants reported working toward a weight‐loss goal, compared with 34.6% of control group participants.

**TABLE 4:** Weight‐management strategies by treatment allocation at baseline and 12 months.

Variables	Baseline			12 mo			Texts with incentives vs. control, OR (97.5% CI)	Texts alone vs. control, OR (97.5% CI)
	Texts with incentives (*n* = 196)	Texts alone (*n* = 194)	Control (*n* = 195)	Texts with incentives (*n* = 145)	Texts alone (*n* = 128)	Control (*n* = 153)		
How often do you keep track of your weight by weighing yourself? *n/N* (%)^a^								
Never	28/194 (14.4)	38/192 (19.8)	30/193 (15.5)	16/141 (11.3)	16/127 (12.6)	19/151 (12.6)	2.2 (1.3–3.5)	1.2 (0.7–1.9)
Less than once/mo	59/194 (30.4)	59/192 (30.7)	57/193 (29.5)	14/141 (9.9)	33/127 (26.0)	39/151 (25.8)		
Once/mo	29/194 (14.9)	31/192 (16.1)	33/193 (17.1)	31/141 (22.0)	32/127 (25.2)	36/151 (23.8)		
Once/wk	50/194 (25.8)	41/192 (21.4)	53/193 (27.5)	40/141 (28.4)	27/127 (21.3)	34/151 (22.5)		
A few times/wk	21/194 (10.8)	18/192 (9.4)	12/193 (6.2)	24/141 (17.0)	15/127 (11.8)	13/151 (8.6)		
Every day	7/194 (3.6)	5/192 (2.6)	8/193 (4.1)	16/141 (11.3)	4/127 (3.1)	10/151 (6.6)		
How often do you monitor your steps? *n/N* (%)^a^								
Never	51/147 (34.7)	49/143 (34.3)	34/144 (23.6)	38/142 (26.8)	31/126 (24.6)	45/151 (29.8)	1.2 (0.7–2.2)	1.7 (0.9–3.1)
Less than once/mo	6/147 (4.1)	15/143 (10.5)	11/144 (7.6)	13/142 (9.2)	18/126 (14.3)	8/151 (5.3)		
Once/mo	5/147 (3.4)	6/143 (4.2)	8/144 (5.6)	5/142 (3.5)	6/126 (4.8)	7/151 (4.6)		
Once/wk	6/147 (4.1)	7/143 (4.9)	8/144 (5.6)	6/142 (4.2)	5/126 (4.0)	11/151 (7.3)		
A few times/wk	30/147 (20.4)	21/143 (14.7)	30/144 (20.8)	28/142 (19.7)	17/126 (13.5)	24/151 (15.9)		
Every day	49/147 (33.3)	45/143 (31.5)	53/144 (36.8)	52/142 (36.6)	49/126 (38.9)	56/151 (37.1)		
Strategies used in past 12 mo to lose weight, *n/N* (%)^b^								
Looked up strategies, tips, or plans on how to lose weight	106/196 (54.1)	95/193 (49.2)	106/195 (54.4)	63/145 (43.4)	60/128 (46.9)	52/153 (34.0)	1.6 (0.9–2.8)	2.0 (1.1–3.5)
Avoided certain foods	157/196 (80.1)	147/193 (76.2)	145/195 (74.4)	119/145 (82.1)	88/128 (68.8)	93/153 (60.8)	3.0 (1.6–5.7)	1.4 (0.8–2.5)
Had a weight goal to work toward	71/196 (36.2)	76/193 (39.4)	80/195 (41.0)	95/145 (65.5)	59/128 (46.1)	53/153 (34.6)	4.7 (2.6–8.5)	1.8 (1.0–3.3)
Reminded yourself of the reasons you are trying to lose weight	117/196 (59.7)	120/193 (62.2)	110/195 (56.4)	109/145 (75.2)	75/128 (58.6)	78/153 (51.0)	3.2 (1.8–5.8)	1.3 (0.7–2.4)
Swapped one type of food for another	98/196 (50.0)	94/193 (48.7)	100/195 (51.3)	68/145 (46.9)	49/128 (38.3)	51/153 (33.3)	2.1 (1.2–3.6)	1.4 (0.8–2.5)
Swapped one type of drink for another	94/196 (48.0)	90/193 (46.6)	95/195 (48.7)	55/145 (37.9)	48/128 (37.5)	47/153 (30.7)	1.5 (0.8–2.6)	1.5 (0.8–2.7)
Told others about your weight‐loss goals	71/196 (36.2)	72/193 (37.3)	74/195 (37.9)	64/145 (44.1)	29/128 (22.7)	29/153 (19.0)	3.9 (2.2–7.1)	1.4 (0.7–2.8)
Used a book, website, or app	83/196 (42.3)	78/193 (40.4)	90/195 (46.2)	50/145 (34.5)	40/128 (31.2)	53/153 (34.6)	1.1 (0.6–2.0)	0.9 (0.5–1.7)
Checked the portion size of things you eat	94/196 (48.0)	101/193 (52.3)	108/195 (55.4)	84/145 (57.9)	75/128 (58.6)	74/153 (48.4)	1.7 (1.0–2.9)	1.6 (0.9–2.9)
Kept track of the calorie/nutritional content of the things you eat and drink	78/196 (39.8)	78/193 (40.4)	87/195 (44.6)	59/145 (40.7)	43/128 (33.6)	50/153 (32.7)	1.7 (1.0–3.2)	1.2 (0.6–2.3)
Used a weight‐loss service to help you manage your weight	31/196 (15.8)	30/193 (15.5)	49/195 (25.1)	10/145 (6.9)	5/128 (3.9)	15/153 (9.8)	0.9 (0.3–2.5)	0.5 (0.1–1.8)
Cut down on alcohol	75/196 (38.3)	80/193 (41.5)	87/195 (44.6)	55/145 (37.9)	53/128 (41.4)	56/153 (36.6)	1.3 (0.7–2.4)	1.3 (0.7–2.4)
Increased the amount of physical activity, sport, or exercise that you were doing	136/196 (69.4)	124/193 (64.2)	129/195 (66.2)	97/145 (66.9)	77/128 (60.2)	79/153 (51.6)	1.9 (1.1–3.4)	1.4 (0.8–2.6)
None	10/196 (5.1)	12/193 (6.2)	9/195 (4.6)	1/145 (0.7)	1/128 (0.8)	12/153 (7.8)	0.1 (0.0–0.8)	0.1 (0.0–1.1)
Another strategy	78/167 (46.7)	56/160 (35.0)	62/162 (38.3)	33/115 (28.7)	25/98 (25.5)	36/128 (28.1)	1.1 (0.6–2.2)	0.9 (0.4–1.8)

Abbreviation: OR, odds ratio.

^a^ Scores range from 1 to 6 (never to every day). Each outcome analyzed using an ordered logit model.

^b^ Each outcome analyzed using a binomial generalized linear model.

Fourteen of the fifteen weight‐loss strategies investigated showed no differences between the texts alone and control groups (Table 4). Compared with the control group, participants in the texts alone group were more likely to report looking up strategies, tips, and plans on how to lose weight (OR 2.0 [97.5% CI: 1.1–3.5]).

#### Weight‐management–related confidence

Compared with the control group, participants in the texts with incentives group had higher levels of confidence in their ability to lose weight (adjusted mean difference = 0.6 [97.5% CI: 0.2–1.0]) and maintain weight loss (adjusted mean difference = 0.9 [97.5% CI: 0.5–1.3]; Table 5). There were no differences in confidence for weight loss and weight‐loss maintenance between the texts alone and control group.

**TABLE 5:** Confidence in weight‐management abilities by treatment allocation at baseline and 12 mo.

	Baseline			12 mo			Texts with incentives vs. control	Texts alone vs. control
Variables	Texts with incentives (*n* = 196)	Texts alone (*n* = 193)	Control (*n* = 195)	Texts with incentives (*n* = 145)	Texts alone (*n* = 128)	Control (*n* = 152)	MD (97.5% CI)	MD (97.5% CI)
Weight‐loss confidence, mean (SD);^a^ *n*	4.2 (1.6); 195	4.3 (1.5); 193	4.5 (1.6); 195	4.7 (1.6); 144	4.3 (1.7); 127	4.1 (1.8); 152	0.6 (0.2 to 1.0)	0.2 (−0.3 to 0.6)
Weight‐loss maintenance confidence, mean (SD);^a^ *n*	3.3 (1.6); 193	3.4 (1.6); 191	3.4 (1.6); 195	4.3 (1.7); 145	3.8 (1.8); 128	3.4 (1.6); 152	0.9 (0.5 to 1.3)	0.3 (−0.1 to 0.7)

Abbreviation: MD, adjusted mean difference.

^a^ Scores range from 1 to 7 (not confident to very confident). Each outcome was analyzed using a linear regression model adjusting for baseline.

## DISCUSSION

This secondary exploratory analysis found little evidence of any clinically important socioeconomic, health, or behavioral moderators of effectiveness for the intervention effects. Hence, Game of Stones appears to be equally effective across a variety of different subpopulations within the trial when examining a multitude of prespecified factors that have been associated with obesity. Based on these findings, the Game of Stones trial interventions of either behavior‐focused text messages alongside financial incentives or text messages alone are unlikely to contribute to intervention‐generated inequalities [22]. This finding is in line with a systematic review examining inequalities in the uptake of, adherence to, and effectiveness of behavioral weight‐management interventions in adults, which found that most trials did not display an inequalities' gradient [20]. However, it should be noted that most trials in this systematic review were unlikely to have sufficient statistical power to identify whether inequalities were present.

Compared with other group‐based, in‐person weight‐management interventions for men living with obesity [8, 9, 10], Game of Stones provides a low‐burden behavioral weight‐management intervention. The current study suggests that both Game of Stones intervention groups may be equally effective in vulnerable groups, including underserved populations of men from lower socioeconomic groups. Furthermore, no evidence of differential effectiveness across a variety of health variables emerged, suggesting that specific targeting of subgroups of men living with obesity is not required to increase intervention effectiveness.

Future research might further examine how to identify individuals who are particularly likely to benefit from the intervention components beyond the socioeconomic and health factors focused on in the current study. Moreover, evidence has suggested that text message–delivered weight‐management support is acceptable and feasible for delivery to postpartum women living with overweight and obesity [42]. Further research could focus on adapting the intervention to women beyond the childbirth context, particularly focusing on those from disadvantaged backgrounds.

There was evidence of participants engaging in several evidence‐based weight‐management strategies, particularly in the texts with financial incentives group. Engagement in strategies to facilitate behavior change is critical for the long‐term maintenance of behavior change and weight [43]. Participants in the texts with financial incentives group reported engaging in more weight‐management strategies, including motivational (e.g., reminding oneself of the reasons for trying to lose weight) and action‐focused strategies (e.g., swapping one type of food for another). Of note is that participants provided with financial incentives reported more goal setting strategies compared with control participants. All participants in this study were provided with a personalized weight‐loss goal following baseline measures by calculating the weight loss required for 5% and 10% weight loss, and it appears that the provision of financial incentives may have increased the relevance of the goal. Moreover, participants in the texts with incentives group reported higher levels of weight‐management confidence compared with the control group, suggesting that incentives alongside behavior‐focused text messages might activate a variety of psychological processes in addition to merely increasing motivation.

Previous evidence has shown that adherence to behavioral weight‐management components is associated with increased weight loss in remotely delivered interventions [44]. Although the current intervention components encouraged self‐tailoring of weight‐loss strategies based on personal preference and circumstance, future research should examine how adherence to key strategies may be optimized, particularly in the texts alone group.

There was no evidence of significant changes in self‐reported health‐related behaviors of physical activity, sedentary behavior, alcohol consumption, or smoking status, which can affect weight loss. The lack of significant change in physical activity at both the moderate and vigorous level is unexpected, given that some evidence has suggested that men often value the use of activity‐related behaviors for weight management [45, 46]. Website information and text messages highlighted that dietary change is required to lose weight, but participants could choose the behavioral focus most relevant for them. Of relevance, a relatively high proportion of participants reported living with multiple long‐term conditions and/or a disability compared with other studies, and these conditions may pose additional challenges for physical activity and attending health promotion services.

The current study has several strengths. The comprehensive moderator analyses undertaken were all prespecified and focused on several relevant factors that might potentially explain differential effects in important subgroups. Moreover, the sample recruited to this trial represents an underserved population of men displaying high levels of obesity, socioeconomic disadvantage, and obesity‐related comorbidities.

This study has some limitations. The sample size considerations for this study are based on changes in the primary outcome weight change at 12 months only, and the current analyses were not considered. This exploratory study presents multiple subgroup and exploratory analyses increasing the chance of type I errors. However, we have some confidence in the largely null findings because the CI values suggest that we are not likely to be missing a clinically important effect size difference among the compared subgroups. Some of the subgroup classifications might have not been optimal, particularly when categorizing continuous variables. Several subgroup analyses (e.g., EQ‐5D‐5L or alcohol consumption) had imbalances across the groups. The behavioral measures obtained were all self‐reported and brief to reduce measurement burden and boost study retention, and dietary intake was not measured.

## CONCLUSION

The Game of Stones trial suggests equitable effectiveness for all men living with obesity regardless of socioeconomic, health, or well‐being status. The texts with financial incentives group showed greater engagement in some weight‐management strategies and favorable changes in weight‐management confidence. The implementation of Game of Stones is unlikely to increase health inequalities.

## CLINICAL TRIAL REGISTRATION

ClinicalTrials.gov ISRCTN91974895.

## CONFLICT OF INTEREST STATEMENT

Dr. Pat Hoddinott reported serving as chair or member of Independent Trial Steering Committees unrelated to weight‐management trials and being a member of the National Institute for Health and Care Research (NIHR) School for Primary Care Research Funding panel. Dr. Kate Hunt reported serving as chair of the Health Improvement, Protection and Services Committee of the Chief Scientist Office, Scotland. Dr. Katrina Turner reported serving as a member of the NIHR HTA commissioning board, December 2017 to September 2020. The other authors declared no conflicts of interest.

## Supporting information


**Data S1.** Supporting Information.

## Data Availability

The data that support the findings of this study are available from the corresponding author upon reasonable request.

## References

[1] GBD Obesity Collaborators; Afshin A, Forouzanfar MH, Reitsma MB, et al . Health effects of overweight and obesity in 195 countries over 25 years. N Engl J Med. 2017;377(1):13‐27. doi:10.1056/NEJMoa1614362 28604169 PMC5477817

[2] UK Office for Health Improvement and Disparities . Obesity Profile: short statistical commentary May 2023. Published May 3, 2023. Acessed February 20, 2024. https://www.gov.uk/government/statistics/obesity‐profile‐update‐may‐2023/obesity‐profile‐short‐statistical‐commentary‐may‐2023

[3] National Institute of Diabetes and Digestive and Kidney Diseases . Overweight & obesity statistics. Updated September 2021. Accessed October 2, 2024. https://www.niddk.nih.gov/health-information/health-statistics/overweight-obesity

[4] Robertson C , Archibald D , Avenell A , et al. Systematic reviews of and integrated report on the quantitative, qualitative and economic evidence base for the management of obesity in men. Health Technol Assess. 2014;18(35):v‐vi, xxiii‐xxix:1‐424. doi:10.3310/hta18350 10.3310/hta18350 PMC4781190 24857516

[5] Caperchione CM , Sharp P . Working with men – perspectives on men's health education and behaviour. Health Educ J. 2025;84(3):213‐218.

[6] Robertson C , Avenell A , Boachie C , et al. Should weight loss and maintenance programmes be designed differently for men? A systematic review of long‐term randomised controlled trials presenting data for men and women: the ROMEO project. Obes Res Clin Pract. 2016;10:70‐84. 25937165 10.1016/j.orcp.2015.04.005

[7] Nguyen D , Liu Y , Kavanagh SA , Archibald D . Gender‐sensitive community weight‐loss programmes to address overweight and obesity in men: a scoping review. BMJ Open. 2024;14:e083646. 10.1136/bmjopen-2023-083646 PMC11243212 38991680

[8] Hunt K , Wyke S , Gray CM , et al. A gender‐sensitised weight loss and healthy living programme for overweight and obese men delivered by Scottish premier league football clubs (FFIT): a pragmatic randomised controlled trial. Lancet. 2014;383:1211‐1221. 24457205 10.1016/S0140-6736(13)62420-4 PMC4524002

[9] Maddison R , Hargreaves EA , Jiang Y , et al. Rugby fans in training New Zealand (RUFIT NZ): a randomized controlled trial to assess the effectiveness of a healthy lifestyle program for overweight men delivered through professional rugby clubs. Int J Behav Nutr Phys Act. 2023;20:37. 36978139 10.1186/s12966-022-01395-w PMC10043512

[10] Petrella RJ , Gill DP , Boa Sorte Silva NC , et al. The hockey fans in training intervention for men with overweight or obesity: a pragmatic cluster randomised trial. EClinicalMed. 2024;77:102911. doi:10.1016/j.eclinm.2024.102911 PMC11576405 39568632

[11] Willcox JC , Dobson R , Whittaker R . Old‐fashioned technology in the era of “bling”: is there a future for text messaging in health care? J Med Internet Res. 2019;21:e16630. 31859678 10.2196/16630 PMC6942182

[12] Skinner R , Gonet V , Currie S , Hoddinott P , Dombrowski SU . A systematic review with meta‐analyses of text message‐delivered behaviour change interventions for weight loss and weight loss maintenance. Obes Rev. 2020;21:e12999. 32043809 10.1111/obr.12999

[13] Ananthapavan J , Peterson A , Sacks G . Paying people to lose weight: the effectiveness of financial incentives provided by health insurers for the prevention and management of overweight and obesity ‐ a systematic review. Obes Rev. 2018;19:605‐613. 29266677 10.1111/obr.12657

[14] Sykes‐Muskett BJ , Andrew P , J LR , Armitage CJ . The utility of monetary contingency contracts for weight loss: a systematic review and meta‐analysis. Health Psychol Rev. 2015;9:434‐451. 25933128 10.1080/17437199.2015.1030685

[15] Boonmanunt S , Pattanaprateep O , Ongphiphadhanakul B , et al. Evaluation of the effectiveness of behavioral economic incentive programs for goal achievement on healthy diet, weight control and physical activity: a systematic review and network meta‐analysis. Ann Behav Med. 2023;57:277‐287. 36367428 10.1093/abm/kaac066 PMC10094952

[16] Ladapo JA , Orstad SL , Wali S , et al. Effectiveness of goal‐directed and outcome‐based financial incentives for weight loss in primary care patients with obesity living in socioeconomically disadvantaged neighborhoods: a randomized clinical trial. JAMA Intern Med. 2023;183:61‐69. 36469353 10.1001/jamainternmed.2022.5618 PMC9857219

[17] Macaulay L , O'Dolan C , Avenell A , et al. Effectiveness and cost‐effectiveness of text messages with or without endowment incentives for weight management in men with obesity (Game of Stones): study protocol for a randomised controlled trial. Trials. 2022;23(1):582. doi:10.1186/s13063-022-06504-5 35869503 PMC9306253

[18] Hoddinott P , O'Dolan C , Macaulay L , et al. Text messages with financial incentives for men with obesity: a randomized clinical trial. JAMA. 2024;332(1):31‐40. doi:10.1001/jama.2024.7064 38744430 PMC11094620

[19] McDonald MD , Dombrowski SU , Skinner R , et al. Recruiting men from across the socioeconomic spectrum via GP registers and community outreach to a weight management feasibility randomised controlled trial. BMC Med Res Methodol. 2020;20(1):249. doi:10.1186/s12874-020-01136-2 33023501 PMC7542377

[20] Birch JM , Jones RA , Mueller J , et al. A systematic review of inequalities in the uptake of, adherence to, and effectiveness of behavioral weight management interventions in adults. Obes Rev. 2022;23(6):e13438. doi:10.1111/obr.13438 35243743 PMC9285567

[21] McDonald MD , Hunt K , Sivaramakrishnan H , et al. A systematic review examining socioeconomic factors in trials of interventions for men that report weight as an outcome. Obes Rev. 2022;23(7):e13436. doi:10.1111/obr.13436 35187778 PMC9285916

[22] Lorenc T , Petticrew M , Welch V , Tugwell P . What types of interventions generate inequalities? Evidence from systematic reviews. J Epidemiol Community Health. 2013;67:190‐193. 22875078 10.1136/jech-2012-201257

[23] Dombrowski SU , McDonald M , Van Der Pol M , et al. Game of Stones: feasibility randomised controlled trial of how to engage men with obesity in text message and incentive interventions for weight loss. BMJ Open. 2020;10:e032653. 10.1136/bmjopen-2019-032653 PMC7045214 32102807

[24] Mackenzie RM , Ells LJ , Simpson SA , Logue J . Core outcome set for behavioural weight management interventions for adults with overweight and obesity: standardised reporting of lifestyle weight management interventions to aid evaluation (STAR‐LITE). Obes Rev. 2020;21:e12961. 31756274 10.1111/obr.12961 PMC7050499

[25] O'Neill J , Tabish H , Welch V , et al. Applying an equity lens to interventions: using PROGRESS ensures consideration of socially stratifying factors to illuminate inequities in health. J Clin Epidemiol. 2014;67:56‐64. 24189091 10.1016/j.jclinepi.2013.08.005

[26] Welch VA , Norheim OF , Jull J , Cookson R , Sommerfelt H , Tugwell P . CONSORT‐Equity 2017 extension and elaboration for better reporting of health equity in randomised trials. BMJ. 2017;359:j5085. doi:10.1136/bmj.j5085 29170161

[27] Petkovic J , Duench SL , Welch V , et al. Potential harms associated with routine collection of patient sociodemographic information: a rapid review. Health Expect. 2019;22:114‐129. 30341795 10.1111/hex.12837 PMC6351414

[28] Abel GA , Barclay ME , Payne RA . Adjusted indices of multiple deprivation to enable comparisons within and between constituent countries of the UK including an illustration using mortality rates. BMJ Open. 2016;6:e012750. 10.1136/bmjopen-2016-012750 PMC5128942 27852716

[29] UK Office for National Statistics . GSS harmonisation support. Accessed August 6, 2024. https://analysisfunction.civilservice.gov.uk/government‐statistical‐service‐and‐statistician‐group/gss‐support/gss‐harmonisation‐support/

[30] Scottish Government . Scottish surveys: Core and harmonised questions. Updated March 21, 2025. Accessed August 6, 2024. https://www.gov.scot/publications/scottish-surveys-core-and-harmonised-questions/

[31] Best M , Papies EK . Lower socioeconomic status is associated with higher intended consumption from oversized portions of unhealthy food. Appetite. 2019;140:255‐268. 31082447 10.1016/j.appet.2019.05.009

[32] French D . Financial strain in the United Kingdom. Oxford Econ Pap. 2017;70:163‐182.

[33] Herdman M , Gudex C , Lloyd A , et al. Development and preliminary testing of the new five‐level version of EQ‐5D (EQ‐5D‐5L). Qual Life Res. 2011;20:1727‐1736. 21479777 10.1007/s11136-011-9903-x PMC3220807

[34] Feng YS , Kohlmann T , Janssen MF , Buchholz I . Psychometric properties of the EQ‐5D‐5L: a systematic review of the literature. Qual Life Res. 2021;30:647‐673. 33284428 10.1007/s11136-020-02688-y PMC7952346

[35] Tennant R , Hiller L , Fishwick R , et al. The Warwick‐Edinburgh Mental Well‐Being Scale (WEMWBS): development and UK validation. Health Qual Life Outcomes. 2007;5:63. doi:10.1186/1477-7525-5-63 18042300 PMC2222612

[36] Kroenke K , Spitzer RL , Williams JB , Löwe B . An ultra‐brief screening scale for anxiety and depression: the PHQ‐4. Psychosomatics. 2009;50:613‐621. 19996233 10.1176/appi.psy.50.6.613

[37] Lillis J , Luoma JB , Levin ME , Hayes SC . Measuring weight self‐stigma: the Weight Self‐Stigma Questionnaire. Obesity. 2010;18:971‐976. 19834462 10.1038/oby.2009.353

[38] UK Office for National Statistics . Disability variable: Census 2021. Updated June 27, 2025. Accessed October 17, 2024. https://www.ons.gov.uk/census/census2021dictionary/variablesbytopic/healthdisabilityandunpaidcarevariablescensus2021/disability

[39] Craig CL , Marshall AL , Sjöström M , et al. International Physical Activity Questionnaire: 12‐country reliability and validity. Med Sci Sports Exerc. 2003;35(8):1381‐1395. doi:10.1249/01.MSS.0000078924.61453.FB 12900694

[40] Hartmann‐Boyce J , Aveyard P , Piernas C , et al. Cognitive and behavioural strategies for weight management in overweight adults: results from the Oxford Food and Activity Behaviours (OxFAB) cohort study. PLoS One. 2018;13:e0202072. 30096203 10.1371/journal.pone.0202072 PMC6086460

[41] LeBlanc ES , Patnode CD , Webber EM , Redmond N , Rushkin M , O'Connor EA . Behavioral and pharmacotherapy weight loss interventions to prevent obesity‐related morbidity and mortality in adults: updated evidence report and systematic review for the US Preventive Services Task Force. JAMA. 2018;320(11):1172‐1191. doi:10.1001/jama.2018.7777 30326501 PMC13151892

[42] McGirr C , Rooney C , Gallagher D , et al. Text messaging to help women with overweight or obesity lose weight after childbirth: the intervention adaptation and SMS feasibility RCT. Public Health Res. 2020;8(4):1‐152. doi:10.3310/phr08040 32223118

[43] Hankonen N . Participants' enactment of behavior change techniques: a call for increased focus on what people do to manage their motivation and behavior. Health Psychol Rev. 2021;15:185‐194. 32967583 10.1080/17437199.2020.1814836

[44] Burke LE , Bizhanova Z , Conroy MB , et al. Adherence to self‐monitoring and behavioral goals is associated with improved weight loss in an mHealth randomized‐controlled trial. Obesity. 2025;33:478‐489. 39962997 10.1002/oby.24234 PMC11897847

[45] Hunt K , McCann C , Gray CM , Mutrie N , Wyke S . “You've got to walk before you run”: positive evaluations of a walking program as part of a gender‐sensitized, weight‐management program delivered to men through professional football clubs. Health Psychol. 2013;32:57‐65. 23316853 10.1037/a0029537

[46] Archibald D , Douglas F , Hoddinott P , et al. A qualitative evidence synthesis on the management of male obesity. BMJ Open. 2015;5:e008372. 10.1136/bmjopen-2015-008372 PMC4606385 26459486

